# Response to Lehrer

**DOI:** 10.1093/jnci/djaf340

**Published:** 2026-01-08

**Authors:** Damir Varešlija, Daniela Ottaviani, Leonie Young

**Affiliations:** The School of Pharmacy and Biomolecular Sciences, The Royal College of Surgeons University of Medicine and Health Sciences, Dublin, Ireland; Beaumont RCSI Cancer Centre, Beaumont Hospital, Dublin, Ireland; Endocrine Oncology Research Group, Department of Surgery, Royal College of Surgeons in Ireland, University of Medicine and Health Sciences, Dublin, Ireland; Beaumont RCSI Cancer Centre, Beaumont Hospital, Dublin, Ireland; Endocrine Oncology Research Group, Department of Surgery, Royal College of Surgeons in Ireland, University of Medicine and Health Sciences, Dublin, Ireland; Department of Surgery, Beaumont Hospital, Dublin, Ireland

To the Editor:

We thank Dr Lehrer for his thoughtful correspondence and The Cancer Genome Atlas (TCGA) based analysis of *CDK12*, *MED1*, and *ERBB2* expression.^1^ Dr Lehrer’s findings reinforce the close association between *CDK12* and *MED1* across breast cancer subtypes and extend discussion on the context-dependent *CDK12* dysregulation in HER2-positive and ER-positive/HER2-negative disease.

As noted, CDK12 and MED1 show strong co-expression even independent of ERBB2 copy number. This supports our mechanistic evidence that CDK12 functionally cooperates with MED1 and ER to sustain transcription in advanced ER-positive tumors. Although transcriptomic correlations highlight potential co-regulation, our study demonstrated that CDK12 directly influences ER chromatin recruitment and transcriptional output, and this is critical to understanding the functional hierarchy between CDK12, MED1, and ER signaling.

We agree that the proposed dual model of genomic co-amplification in HER2-positive cancers and transcriptional co-activation in ER-positive/HER2-negative cancers offers a useful framework. Clinical series of HER2-positive breast cancers have similarly shown recurrent *ERBB2*/*CDK12* co-amplification and linked it to therapeutic outcome,^2^^,^^3^ which supports the genomic dosage context described by Dr Lehrer. Moreover, targeting CDK12 in HER2-amplified models reduces PI3K/AKT signaling and restores anti-HER2 sensitivity,^4^^,^^5^ also confirming functional cooperation between CDK12 and HER2 pathways.

Beyond receptor-positive disease, pharmacological studies in triple-negative breast cancer have demonstrated that dual *CDK12/CDK13* inhibition provokes intronic polyadenylation, suppresses DNA-damage-repair gene expression, and induces a “BRCAness” phenotype that sensitizes tumors to PARP inhibitors and platinum agents.^6^^,^^7^

These observations indicate that *CDK12* can regulate distinct transcriptional programs depending on molecular context, including genomic co-amplification in HER2-positive disease, CDK12/MED1-driven co-activation in luminal tumors, and potential DNA-repair gene control in basal-like disease (Figure 1). In practice, distinguishing between these contexts will be essential when designing therapeutic strategies that target the *CDK12* complex, because these mechanisms may not be mutually exclusive.

**Figure 1.**
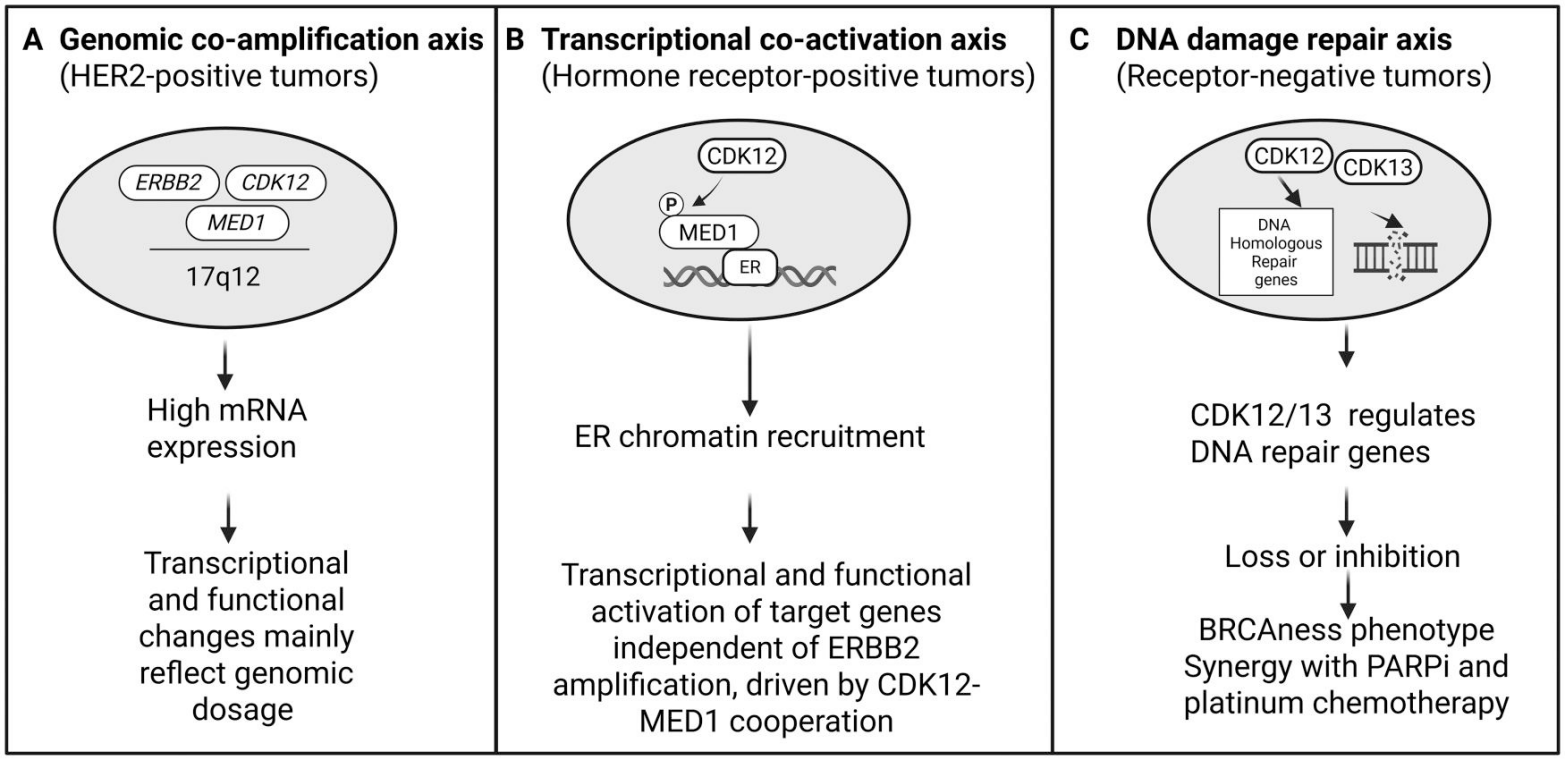
Distinct contexts of *CDK12* dysregulation in breast cancer. **(A)** In HER2-positive tumors, *CDK12* and *ERBB2* are co-amplified on chromosome 17q12, leading to increased mRNA expression primarily reflecting genomic dosage. **(B)** In ER-positive/HER2-negative tumors, CDK12 phosphorylates and activates MED1, facilitating estrogen receptor (ER) chromatin recruitment and transcriptional activation of ER-responsive genes. **(C)** In triple-negative breast cancer, loss or pharmacological inhibition of *CDK12/CDK13* promotes intronic polyadenylation and reduced DNA-damage-repair (DDR) gene expression, resulting in a BRCAness phenotype and enhanced sensitivity to PARP inhibitors and DNA-damaging agents. Figure created with BioRender.com.

## Data Availability

No new data was analyzed as part of this article.
